# The m6A mRNA demethylase FTO in granulosa cells retards FOS-dependent ovarian aging

**DOI:** 10.1038/s41419-021-04016-9

**Published:** 2021-07-27

**Authors:** Zhong-xin Jiang, Yi-ning Wang, Zi-yuan Li, Zhi-hui Dai, Yi He, Kun Chu, Jia-yi Gu, Yi-Xuan Ji, Ning-xia Sun, Fu Yang, Wen Li

**Affiliations:** 1 Center for Reproductive Medicine & Fertility Preservation program, International Peace maternity and Child health Hospital, School of Medicine, Shanghai Jiao Tong University, Shanghai, 200030 China; 2 The Center of Reproductive Medicine, Shanghai Changzheng Hospital, Naval Medical University, Shanghai, 200003 China; 3 Obstetrics and Gynecology Department, The 1st Naval hospital of Southern Theater Command, Guangdong, 524005 China; 4 The Department of Medical Genetics, Naval Medical University, Shanghai, 200433 China

**Keywords:** RNA modification, Infertility

## Abstract

Multifunctional *N*6-methyladenosine (m6A) has been revealed to be an important epigenetic component in various physiological and pathological processes, but its role in female ovarian aging remains unclear. Thus, we demonstrated m6A demethylase FTO downregulation and the ensuing increased m6A in granulosa cells (GCs) of human aged ovaries, while FTO-knockdown GCs showed faster aging-related phenotypes mediated. Using the m6A-RNA-sequence technique (m6A-seq), increased m6A was found in the FOS-mRNA-3′UTR, which is suggested to be an erasing target of FTO that slows the degradation of FOS-mRNA to upregulate FOS expression in GCs, eventually resulting in GC-mediated ovarian aging. FTO acts as a senescence-retarding protein via m6A, and FOS knockdown significantly alleviates the aging of FTO-knockdown GCs. Altogether, the abovementioned results indicate that FTO in GCs retards FOS-dependent ovarian aging, which is a potential diagnostic and therapeutic target against ovarian aging and age-related reproductive diseases.

## Introduction

Driven by the postponement of pregnancy due to socioeconomic and demographic trends over the past several decades, female reproductive aging and ensuing age-related infertility have become increasingly prevalent [1, 2]. In cases of female fertility, reproductive aging has been identified as the single largest irreversible contributor to compromised gamete quality and one that remains recalcitrant to current therapeutic interventions [3, 4]. In fact, the female reproductive system, especially the ovary, is particularly vulnerable to age-associated changes, and the ovary is one of the organs with early-onset aging-associated dysfunction with obvious functional declines after the age of 30 years [5, 6], which impairs the stable sexual hormone environment and normal ovulation function. However, our understanding of this process is insufficient at present. It is of great practical significance to explore the pathophysiological mechanism of female ovarian aging and the pathogenesis of female infertility.

The follicle is the basic functional unit of the ovary and it is comprised of a variety of heterogeneous cells. Granulosa cells (GCs) surrounding the oocyte, as important supporting cells, have multiple complex spatiotemporal connections to oocytes and the internal environment outside follicles [7]. The development of follicles and the maturation of oocytes depend on oocyte-GC mutual regulatory signaling pathways and a wide range of molecular regulatory mechanisms [8]. GCs are one of the major participants in follicular initiation, recruitment, selection, dominance, ovulation, and luteinization, and they play an important role in early-onset ovarian dysfunction, polycystic ovarian syndrome, and some other pathological processes [9]. They are decisive as to the fate of follicles and are also signal transducers and responders to aging-related factors that affect follicles [10]. It is widely recognized that abundant epigenetic changes occur in GCs, affecting the development of the oocytes during the ovarian aging process [11, 12], such as DNA methylation and histone changes [13]. Previous studies indicated that it is of great significance to explore the epigenetic changes of GCs and the subsequent molecular biological mechanism behind them in the ovarian aging process [14, 15]. However, the posttranscriptional regulation of gene expression involved in the ovary aging process has not yet been fully investigated.

*N*6-methyladenosine (m6A) is a ubiquitous and conserved posttranscriptional epigenetic modification in diverse regions of different RNAs [16]. Similar to other epigenetic modifications, such as DNA methylation, modification of m6A is dynamic and reversible, created by the METTL3–METTL14-WTAP methyltransferase complex and erased by demethylases, including the fat mass and obesity-associated protein (FTO) and the AlkB homolog 5 RNA demethylase (ALKBH5) [17]. Multiple biological functions of m6A, including RNA metabolism, mRNA stability, translation efficiency, alternative splicing, and cytoplasmic mRNA turnover [18, 19], have been revealed under various extensive normal physiological and pathological conditions, including tumorigenesis, metabolism, senescence, animal development, and stem cell fate determination [18]. Of note, several studies have revealed that the spatiotemporal differences and many related pathways of m6A during reproductive development are involved in gamete meiosis [20, 21], maternal zygote transformation [22, 23], and embryonic stem cell differentiation [24, 25]. However, many aspects of RNA m6A modification in reproductive physiology and pathology, especially during the ovarian aging process, have not been fully elucidated, which attracted our attention.

Therefore, we designed this study to determine the role of m6A in the ovarian aging process. An increasing total m6A content was revealed in GCs of aged ovaries, and changes in the expression level of the key modification enzyme, namely, downregulation of demethylase FTO were observed, the key downstream target gene of which was identified as FOS, a powerful transcription factor subunit and aging-related protein. Increased m6A in the FOS-mRNA-3′UTR impairs the normal degradation process of FOS-mRNA, which subsequently results in the aging of GCs due to increased levels of FOS.

## Results

### The abnormally increased m6A modification in GCs of aged ovaries was due to the downregulation of the demethylase FTO

**Fig. 1:**
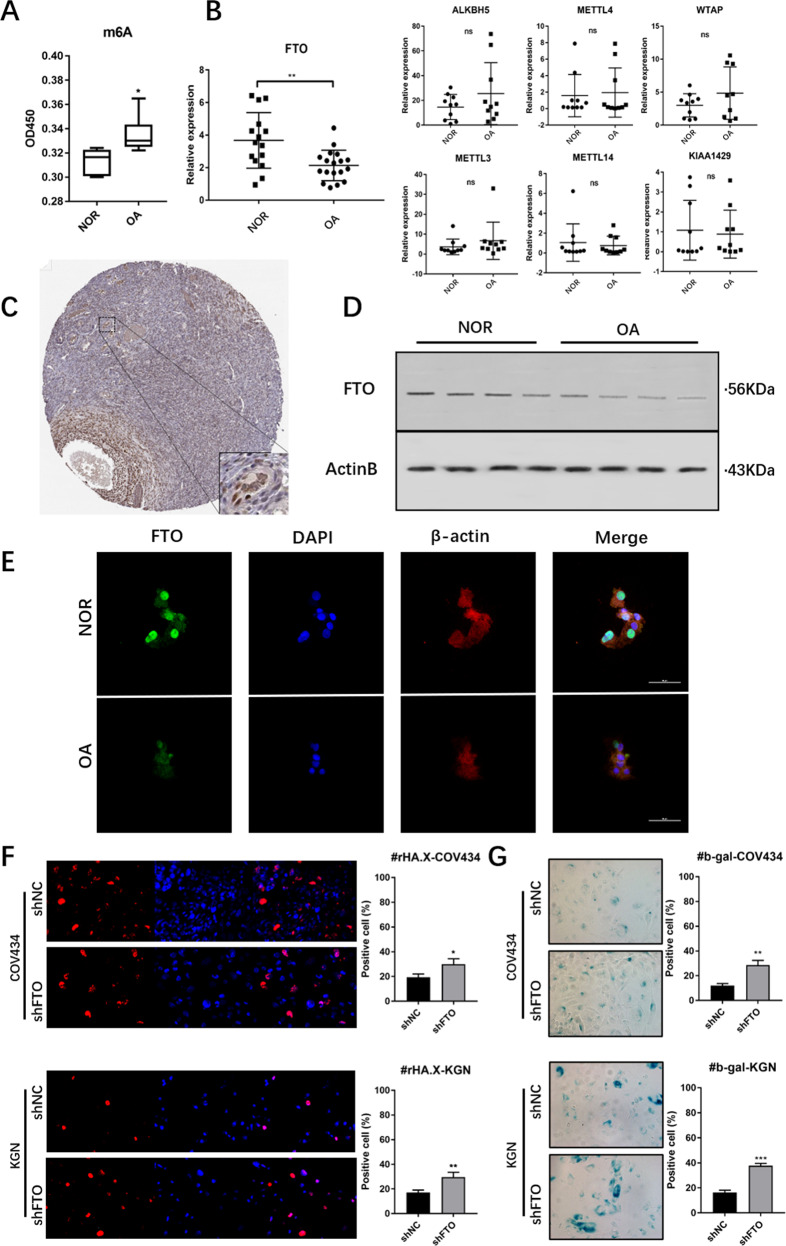
Levels of RNA m6A and expression of FTO in GCs of OA patients and controls. **A** Increased m6A modification in GCs of aged ovary (OA) compared to the normal ovarian reserve group (NOR), *n* = 6. **B** RT-PCR shows the mean expression levels of m6A-associated genes in the GCs in OA and NOR; for FTO, *n* = 15; and for other genes, *n* = 10. **C** Immunohistochemistry (from www.proteinatlas.com) showed the specific expression of FTO in granulosa cells of ovarian tissue. **D**, **E** Western blot and immunofluorescence analyses showing the downregulation of FTO in aged granulosa cells compared to young granulosa cells. **F** Immunofluorescence of γH2A. X and **G** β-galactosidase staining in FTO-silenced KGN and COV434 cells more readily enter senescence induced by hydrogen peroxide. All experiments were performed three times. The error bars indicate SD; *p < 0.05, **p < 0.01, ***p < 0.001.

To detect the correlation between the aging process of the ovary and the m6A modification, the total m6A modification of the GCs of six pairs of aged ovaries and control ovaries was detected by colorimetry, and the quantity of m6A was significantly higher in the total RNA of GCs in aged ovaries than in normal ovaries (Fig. 1A). Because m6A is written/erased by a series of methylases/demethylases, such as METTL3, METTL4, METTL14, WTAP, KIAA1429, ALKBH5, and FTO, to identify which gene mainly causes the increase in the m6A, real-time PCR (RT-PCR) was performed. The results showed that the expression of FTO was downregulated in the GCs of aged ovaries, while the expression of METTL3, METTL4, METTL14, WTAP, and KIAA1429 were not significantly different (Fig. 1B), and it was found that FTO had a specific high expression in GCs by querying the proteinatlas database (https://www.proteinatlas.org/) (Fig. 1C). Western blotting (Fig. 1D) and immunofluorescence (Fig. 1E) were used to detect the protein level of FTO in GCs, and the expression of FTO was decreased in the GCs of aged ovaries. To verify the relationship between FTO and aging in GCs, COV434-shFTO, KGN-shFTO, and their negative controls COV434-shNC and KGN-shNC were constructed in a lentivirus expression system in the COV434 and KGN cell lines (Supplementary Fig. S1). Dot blot analysis showed increased m6A modification in FTO knockdown GCs (Supplementary Fig. S2).

To determine whether FTO knockdown could mediate the senescence of GCs, a medium containing 50 µm hydrogen peroxide was used to induce the senescence process. Compared with the negative control, the expression of γ H2A. X, a marker of senescence, was increased (Fig. 1F), and β-galactosidase staining was also increased (Fig. 1G) in COV434-shFTO and KGN-shFTO cells. All of the above results suggested that FTO in GCs might play an important role in the ovarian aging process.

### Methylated RNA immunoprecipitation sequencing revealed potential m6A erasing targets of FTO in granulosa cells

**Fig. 2:**
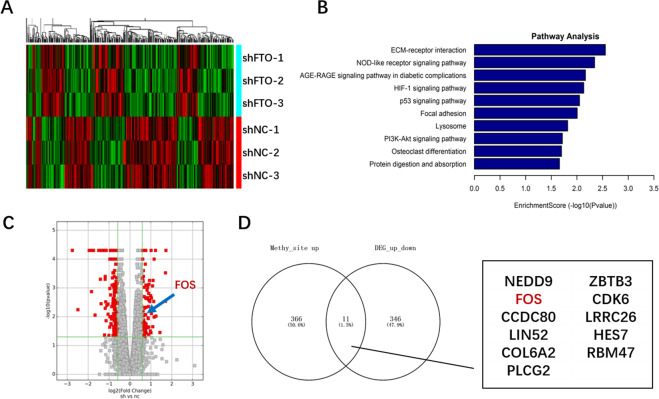
MeRIP-seq and mRNA-seq of COV434-shFTO and COV434-shNC. **A** Clustered heat map of the differentially expressed mRNAs in COV434-shFTO (shFTO1-3) and COV434-shNC (shNC1-3) cells. Rows represent cells while columns represent mRNAs. **B** GO analysis of differentially expressed mRNAs in COV434-shFTO and COV434-shNC. **C** Volcano plot showing differentially expressed mRNAs between COV434-shFTO and COV434-shNC in red dots. FOS is indicated by arrows (fold change >2.0 and *P* < 0.01). **D** Venn diagram illustrating the overlap in differential m6A modifications and expression. Eleven genes were found to be possible targets of FTO.

To explore the downstream m6A erasing targets by FTO, methylated RNA immunoprecipitation sequencing (MeRIP-seq) and transcriptome sequencing (mRNA-seq) were performed in three repeats of the stable FTO-knockdown COV434-shFTO and COV434-shNC. According to quantile normalized statistical analysis of the data obtained, 545 peaks of 366 genes with upregulation of methylation were screened out with a fold change cutoff greater than 2 and a *p* value cutoff less than 0.00001, and 346 differential genes were identified with a fold change cutoff greater than 1.5 and a *p* value cutoff of less than 0.05. Heat map analysis showed that differentially expressed genes were clustered, and there were significant differences between the two groups (Fig. 2A). GO analysis showed that the differentially expressed genes were enriched in the ECM-receptor interaction, the NOD-like receptor signaling pathway, and the AGE-RAGE signaling pathway (Fig. 2B), which are related to the function of GCs or the aging process [10, 26, 27]. Furthermore, a volcano map showed the differentially expressed genes in red (Fig. 2C). Finally, 11 significantly changed genes overlapped in MeRIP-seq and mRNA-seq of FTO-knockdown COV434, which is shown in a Venn diagram (Fig. 2D).

### FTO downregulation increased m6A modification of FOS mRNA and upregulated the expression of FOS

**Fig. 3:**
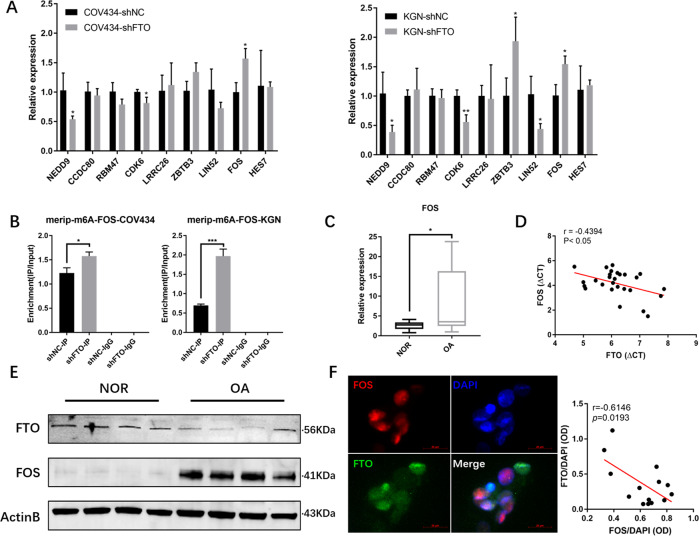
FOS is the m6A erasing target of FTO in GCs. **A** RT-PCR showed the relative expression of 11 potential target genes in COV434-shFTO/COV434-shNC and KGN-shFTO/KGN-shNC. **B** Immunoprecipitation of m6A-modified RNA in control or FTO-knockdown cells followed by RT-PCR to assess FOS mRNA m6A modification levels. **C** RT-PCR showed the differential expression of FOS between OA and NOR and **D** the negative correlation between FTO and FOS in GCs. **E** Western blot analysis showed the differential expression of FOS and FTO between OA and NOR. **F** Immunofluorescence showed FTO (green) and FOS (red) located in different parts of the ovarian granulosa cells. All experiments were performed three times, and cells in three random fields were analyzed. The error bars indicate SD; *p < 0.05, **p < 0.01, ***p < 0.001.

Because both groups of experiments point to these 11 genes, which are most likely the target genes of FTO, considering the inherent error of high-throughput sequencing, we further verified the above 11 potential targets. Thus, the 11 molecules were verified again by RT-PCR in FTO knockdown COV434 and KGN cells and the controls (Fig. 3A). The expression levels of COL6A2 and PLCG2 in the two kinds of cells were low, which is not shown in the figure, while FOS, NEDD9, and CDK6 were significantly different. In addition, in the two cell lines that overexpressed FTO, FOS was found to be significantly downregulated (Supplementary Fig. S3). MeRIP-qPCR confirmed that m6A modification of FOS mRNA was relatively increased in COV434-shFTO and KGN-shFTO cells compared with their negative controls (Fig. 3B). In addition, RIP-qPCR was performed with an FTO antibody and we found that the FTO protein could bind to FOS mRNA (Supplementary Fig. S4) to erase the m6A modification.

Then, we explored the expression abundance of FOS and m6A and their relationship with FTO in COV434 and KGN cells, which still needs to be verified in human ovarian GCs. Therefore, we detected the RNA expression level of FOS in clinical GC samples of aging ovaries and normal ovarian reserve samples and found that FOS was highly expressed in GCs of aged ovaries (Fig. 3C) and was negatively correlated with the expression level of FTO (Fig. 3D). Detection of FOS protein expression levels showed the same expression trend (Fig. 3E). Immunofluorescence was used to detect the expression of FTO and FOS in human GCs, and it was also found that their expression showed a negative correlation in different cell types (Fig. 3F).

### The increase in m6A modification in the 3′UTR resulted in reinforcing the stability of FOS-mRNA

**Fig. 4:**
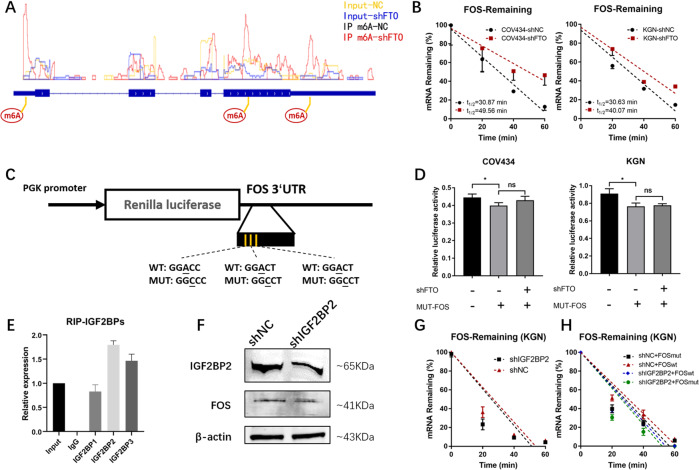
The increase in m6A modification in the 3′UTR resulted in increased stability of the FOS mRNA. **A** MeRIP-seq showed the different m6A peaks of FOS mRNA in COV434-shFTO compared to COV434-shNC. IP immunoprecipitation. **B** Transcriptional inhibition experiments showed a longer half-life of FOS mRNA in FTO-knockdown COV434 and KGN cells. **C** Wild-type or m6A consensus sequence mutant FOS-3′UTR cDNA was fused behind the Renilla luciferase reporter. **D** Relative luciferase activity of the wild-type and mutant FOS 3′UTR reporter vectors in FTO-knockdown COV434 and KGN cells. **E** RIP-qPCR showed the combination of IGF2BPs antibodies and FOS-mRNA. **F** Western blots showed lower FOS expression in IGF2BP2-knockdown KGN compared with negative control. **G** Transcriptional inhibition experiments showed a shorter half-life of FOS mRNA in IGF2BP2-knockdown KGN cells. **H** Transcriptional inhibition experiments of wild-type and mutant FOS 3′UTR reporter vectors in IGF2BP2-knockdown (and negative control) KGN cells. All experiments were performed three times. The error bars indicate SD; *p < 0.05.

FOS has been proven to be an important target of the demethylase FTO in the modification of m6A in GCs, and the m6A modification of GGAC consensus sequences in the 3′UTR is thought to affect the stability of the mRNA [28]. At the same time, the location of the 3 sequence context of the GGAC in the FOS-mRNA-3′UTR was consistent with the m6A modification peak of the 3′UTR, which was found by MeRIP-seq (Fig. 4A). Therefore, actinomycin D was used to inhibit transcription to investigate the difference in the degradation time of FOS mRNA within the COV434-shFTO, KGN-shFTO, and their respective control groups. The decay of FOS mRNA in FTO knockdown cells was significantly slower than that in FTO control cells (Fig. 4B). To further address the effect of m6A modification on FOS expression, we performed sequence-based RNA adenosine methylation site prediction by SRAMP [29] in FOS-mRNA (Supplementary Table 1) and constructed both wild-type (WT) and mutant (MUT) FOS reporter minigenes. The adenosine bases of the predicted RNA adenosine methylation sites in the FOS-mRNA-3′UTRs were replaced by cytosine to abolish the m6A modification (Fig. 4C). Compared to the MUT, the luciferase activity of the WT FOS-3′UTR-fused reporter was significantly augmented upon FTO silencing, while FTO knockdown showed no effect on the expression of the MUT FOS-fused reporter, suggesting that the modulation of FOS expression was under the control of FTO-associated m6A modification (Fig. 4D).

The GGAC sequence in the 3′UTR region of FOS mRNA was proved to be the target by the above experiments. Considering insulin-like growth factor 2 mRNA-binding proteins (IGF2BPs) as a distinct family of m6A readers enhances mRNA stability through recognizing the consensus GG(m6A)C sequence was reported [30]. IGF2BPs were subsequently verified that can combine with FOS mRNA (Fig. 4E). Considering the combining ability to FOS mRNA, IGF2BP2 was chosen to be knockdown in KGN and decreased FOS expression (Fig. 4F), as well as faster decay of FOS-mRNA (Fig. 4G), was observed. To explore the relationship between IGF2BP2 and GG(m6A)C on FOS-mRNA-3′UTR, the above WT and MUT FOS reporter minigenes were used in IGF2BP2-knockdown and negative control cells (Fig. 4H). Both IGF2BP2 knockdown and MUT (compared with WT) accelerated the degradation process, while the degradation rate of MUT was not so significantly different(compared with that of negative control of IGF2BP2) from that of WT after IGF2BP2 knockdown. The differences that still exist may be because of the non-knockdown of IGF2BP1 and IGF2BP3.

### Silencing FOS partially alleviated FTO-dependent aging

**Fig. 5:**
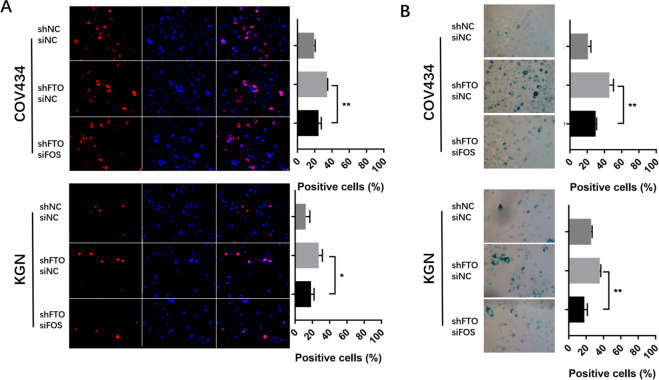
Silencing FOS partially alleviated FTO-dependent aging in COV434 and KGN cells. Immunofluorescence of γH2A. X (**A**) and β-galactosidase staining (**B**) in FTO-silenced and FOS-silenced KGN and COV434 cells more readily entered senescence induced by hydrogen peroxide. All experiments were performed three times. The error bars indicate SD; *p < 0.05, **p < 0.01, ***p < 0.001. All experiments were performed three times, and three microscopic images were counted in each group. The error bars indicate SD; *p < 0.05, **p < 0.01, ***p < 0.001.

To investigate whether the abnormal upregulation of FOS is the key node of the GC senescence phenotype caused by FTO downregulation, we transferred FOS siRNA into COV434 and KGN cells before and after FTO knockdown (Supplementary Fig. S5). It was found that the ROS-related senescence phenotype of GCs was partially restored after FOS siRNA transfer (Fig. 5A, B), which indicated that FOS is the downstream target of demethylase FTO after performing its function by erasing m6A and it is the key node, causing GC aging through m6A modification.

### ROS may initiate the FOS-dependent ovarian aging process induced by decreasing FTO

**Fig. 6:**
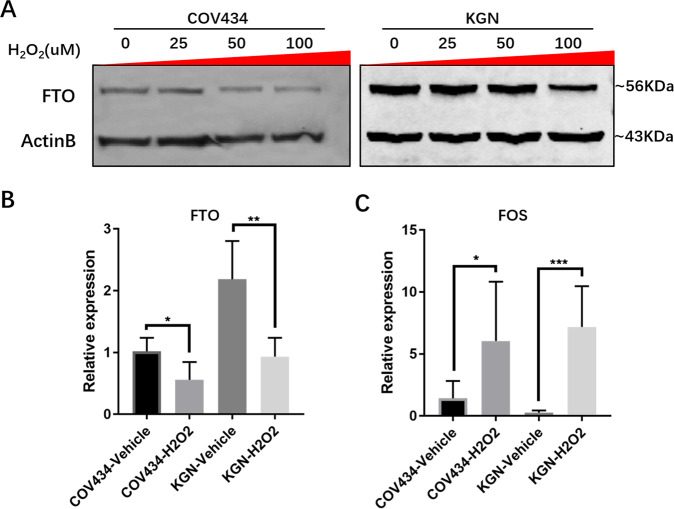
ROS may be the cause of abnormal FTO levels and m6A disorder in aged ovarian granulosa cells. **A** Western blot analysis showed that FTO was downregulated with increasing levels of hydrogen peroxide. **B** RT-PCR showed the downregulation of FTO by hydrogen peroxide. **C** The effect of hydrogen peroxide on the expression of FOS in GCs was shown by RT-PCR. Performed three times. The error bars indicate SD; *p < 0.05, ***p < 0.001.

Previous studies have shown that ROS is an important factor leading to aging [31]. In the process of primate ovarian aging, a decline in antioxidant capacity is also one of the main characteristics [14, 32]. Therefore, we speculated that ROS might be the driving force for the decrease in FTO levels in GCs. The protein abundance (Fig. 6A) and RNA expression (Fig. 6B) of FTO decreased after incubation with hydrogen peroxide as the source of ROS in COV434 and KGN cells. In addition, the m6A modification of total RNA (Supplementary Fig. S6) and the RNA expression of FOS (Fig. 6C) significantly increased after incubation with hydrogen peroxide. Altogether, ROS may initiate the FOS-dependent ovarian aging process induced by decreasing the level of FTO in GCs.

## Discussion

Our study is the first report on the m6A-related pathway in GCs in the ovarian aging process. Specifically, this study makes three noteworthy contributions. First, an m6A modification disorder in GCs was found in the ovarian aging process of humans, and downregulation of the demethylase FTO was identified to be the key factor behind the m6A modification disorder. Second, FTO regulates the expression of FOS through an m6A-related pathway, which was identified as the FTO-FOS axis and is important in the ovarian aging process. Third, the correlation between aging-related factors and the m6A-related pathway was explored, which was complemented by the epigenetic pathway of aging. This implies that the m6A-related pathway between FTO and FOS may be a potential therapeutic target for ovarian aging.

The role of m6A in reproduction, including gametogenesis, embryo development, and the ovarian aging process, has been gradually revealed. It has been reported that the downregulation of FTO mediates the development of male asthenospermia through m6A modifications [33]. Interestingly, several studies prior to the discovery of m6A indicated that FTO is closely related to obesity, telomere length, and other aging factors [34]. Recently, a study showed that FTO is necessary for the maintenance of bone mass and functions to protect osteoblasts from the ROS-mediated cell aging process through the m6A pathway [35], which coincides with our research findings. Our results showed that FTO is the key enzyme for increasing m6A modifications in aged ovaries. In our study, an upregulation trend was presented, although the difference in expression of METTL3 in the GCs of aged ovaries was not statistically significant. METTL3 has been shown to affect the differentiation of spermatogonia and the initiation of meiosis associated with asthenospermia [33, 36]. However, the effect of m6A on ovarian aging has not been reported, and the underlying mechanisms have not been clearly explained, which is why we performed this study. Remarkably, a previous study reported that a decrease in FTO in the GCs of patients with premature ovarian insufficiency increased the total amount of m6A [37], while another study reported that alkylating agents could increase the abundance of m6A modifications in GCs [38]. These results support the findings of our study because premature ovarian insufficiency is a subclass of ovarian dysfunction possibly caused by ovarian aging [39, 40].

FOS, as a subunit of the transcription factor activator protein 1(AP-1), is generally known to regulate multiple life processes and is multifunctional in different tissue types according to neuroscience and tumor-related studies [41]. Among aging-related fields, FOS can mediate UV-related skin aging by upregulating matrix metalloproteinases (MMPs) to downregulate TIMP [42–44]. In a recent study[45], FOS/AP-1 demonstrated “pioneers” the senescence enhancer landscape by change the epigenetic state of enhancers to trigger genome-wide enhancer reprogramming and to triggers senescence-associated secretory phenotype (SASP), resulting in senescent cell fate. It has been reported that aging factors such as ROS [46] and homocysteine [47] increase the expression of FOS in some other cells, while we shed light on the relationship between ROS, FTO, and FOS by m6A modification in GCs. At the same time, it is interesting that high expression of FOS can be detected in FTO knockdown MDA-MB-231 and MCF-4 cells [48], which is worthy of further verification and indicates that the process by which FTO regulates FOS through the m6A pathway may also occur in breast cancer or other kinds of cells or tissues. In addition, a lack of FOS disrupts ovulation processes by inducing an ovulatory prostaglandin disorder and it impairs luteal function and the sexual hormonal environment [49–51]. According to our results, we speculate that FOS-dependent changes in GCs may lead to abnormal ovulation processes, incoordination between oocytes and ovulation processes, overmaturation or limited maturation of oocytes, and damage to their developmental potential [52], which has been confirmed by clinical reports [53, 54] and animal studies [55, 56]. Thus, the role of FOS warrants further exploration in ovarian aging.

Although the FTO-FOS axis was first reported as an m6A-related pathway in the ovarian aging process within humans by our study and GCs collected from ART patients were used in our experiments, the application of our results in clinical diagnosis and treatment still has a long way to go. GCs are often discarded as waste products during oocyte retrieval, and what we strive to do is turn this waste into treasure. In fact, we are advancing a clinical trial to assess the feasibility of the FTO-FOS axis for predicting ovarian reserve and single oocyte quality, by which we hope to guide laboratory operations of ART. In recent years, progress has been made in the exploration of possible anti-ovarian aging agents or approaches [57]. However, the effects of antioxidants, calorie restriction, simulants, or autophagy inducers are still unsatisfactory and limited, and other drugs still need to be developed. As one of the potential targets, RNA epigenetic modifications such as m6A are worth developing and utilizing.

**Fig. 7:**
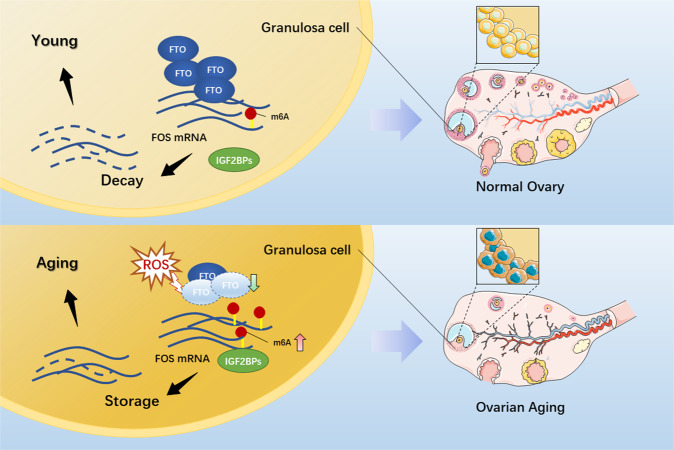
Working model. The role of FTO as an m6A demethylase in ovarian aging. Aging-related factors (ROS) downregulate the demethylase FTO in ovarian granulosa cells, which can enhance FOS-mRNA stability by increasing m6A in the FOS-mRNA-3′UTR to upregulate FOS expression, thus leading to ovarian aging.

In summary, a possible epigenetic mechanism of the ovarian aging process was identified by our study (Fig. 7). In the process of ovarian aging, the accumulation of ROS results in the low expression of the demethylase FTO in GCs, and the ensuing increase in m6A in the FOS-mRNA-3′UTR slows the degradation of FOS-mRNA to upregulate FOS in GCs, promoting the aging of GCs and ultimately accelerating ovarian aging. In addition, this evidence of the relationship between m6A in GCs and ovarian aging expands the view of ovarian aging and establishes new targets for developing interventions against physiological (and/or premature) ovarian aging and the ensuing female reproductive dysfunction and for developing routines for aged ovary rejuvenation and female fertility preservation.

## Materials and methods

### Human subjects and GC sample preparation

This study was approved by the ethics committee of Changzheng Hospital Affiliated with Naval Medical University. With the informed consent of the patients, GC samples from in vitro fertilization (IVF) or intracytoplasmic sperm injection (ICSI) treatment (IVF-ICSI) patients were collected after controlled ovarian hyperstimulation at the reproductive medicine center of the Changzheng Hospital Affiliated with the Naval Medical University, while the preparation of GC samples in the laboratory followed our procedure reported previously [58]. Because of the method of samples collecting, most of the cells of the GC samples from patients are actually cumulus cells. The case eligibility and exclusion criteria of ovarian aging were based on the Bologna criteria for poor ovarian response published by the European Society of Human Reproduction and Embryology (ESHRE) in 2011, with reference to the POI clinical guidelines published by ESHRE in 2016 and several perspectives on the definition of ovarian aging. More than two reproductive specialists determined whether patients were enrolled according to those criteria (Supplementary Table 2). The major clinical endocrine parameters and anthropometric variables of the enrolled subjects are presented in Supplementary Table 3.

### Cell cultures

The human ovarian granulosa cell lines KGN and COV434 with STR (short tandem repeat) identification were cultured in DMEM (HyClone, Logan, UT, USA) containing 10% fetal bovine serum (FBS) (Gibco, Detroit, MI, USA) and 100 IU/mL penicillin-streptomycin in a humidified atmosphere of 5% CO2 and 95% air at 37 °C. The STR sites of the two kinds of cells are shown in Supplementary Table 4.

### RNA m6A quantification

This experiment was carried out with the m6A RNA Methylation Assay Kit (Colorimetric; Abcam, Cambridge, USA). Total RNA extracted with RNAiso Plus (TAKARA, Beijing, China) was treated with DNase I (Promega) and then detected with a NanoDrop 2000 spectrophotometer. The samples were diluted to the required concentration with RNase-free water. All other steps were performed according to the instructions of the kit, and the m6A was quantified by reading the absorbance at a wavelength of 450 nm for each well with a multifunctional microplate reader and was calculated based on the standard curve.

### RNA-sequence

Total RNA was extracted with RNAiso Plus (TAKARA, Beijing, China) from stable shFTO COV434 cells and the controls, which was used to remove the rRNAs by Ribo-Zero rRNA Removal Kits (Illumina, San Diego, CA, USA) following the manufacturer’s instructions. RNA libraries were constructed by using rRNA-depleted RNAs with the TruSeq Stranded Total RNA Library Prep Kit (Illumina, San Diego, CA, USA) according to the manufacturer’s instructions. Libraries were controlled for quality and quantified using the BioAnalyzer 2100 system (Agilent Technologies, Inc., USA). Ten picometer libraries were denatured as single-stranded DNA molecules, captured on Illumina flow cells, amplified in situ as clusters, and finally sequenced for 150 cycles on an Illumina HiSeq Sequencer according to the manufacturer’s instructions. Library preparation and high-throughput sequencing were provided by CloudSeq Biotech (Shanghai, China).

### MeRIP-seq

The preparatory work of the RNA m6A-sequence was the same as for the RNA-sequence. For the follow-up procedure, m6A RNA immunoprecipitation was performed with the GenSeqTM m6A RNA IP Kit (GenSeq Inc., China) by following the manufacturer’s instructions. Both the input sample without immunoprecipitation and the m6A IP samples were used for RNA-seq library generation with the NEBNext^®^ Ultra II Directional RNA Library.

### RNA isolation and real-time polymerase chain reaction (RT-PCR)

Total RNA from GCs was extracted with RNAiso Plus (TAKARA, Beijing, China) and reverse-transcribed into cDNA using the High-Capacity RNA-to-cDNA Kit (TAKARA, Beijing, China) with random hexamer primers, while the ABI StepOne Plus system was used to perform RT-PCR to measure duplex DNA formation with SYBR Premix Ex Taq II (TAKARA, Beijing, China). The sequences of the primers used are listed in Supplementary Table 5, and the results were normalized by GAPDH levels.

### Western blot analysis

Cells were lysed in ice-cold radioimmunoprecipitation assay (RIPA) lysis buffer (Beyotime, Shanghai, China) with Protease/Phosphatase Inhibitor Cocktail (Epizyme, Shanghai, China). Equal amounts and volumes of protein were separated by 12.5% SDS–polyacrylamide gel electrophoresis and transferred onto polyvinylidene fluoride membranes (Millipore, USA). The membranes were incubated overnight at 4 °C with diluted primary antibodies (Supplementary Table 6) after blocking with 5% nonfat milk for 1 h and then incubated with IRdye 700-conjugated goat anti-rabbit IgG and/or IRdye 800-conjugated goat anti-mouse IgG. An Odyssey infrared scanner (Li-COR Biosciences, Nebraska, USA) was used to detect the blots, which were normalized to β-actin levels.

### RNA m6A dot blot assay

To conduct the m6A dot blot assay, the indicated amount of total RNA was denatured at 65 °C for 5 min, followed by chilling on ice. Five microliters of RNA (800 ng) were double-diluted and spotted on an Amersham Hybond-N + membrane (GE Healthcare, Piscataway, NJ) for each dot. After UV crosslinking three times, the membrane was washed with PBS and then blocked with Nucleic Acid Blocking Buffer (Thermo Fisher, USA) for 1 h, which was then incubated with anti-m6A antibody (1:1000; Synaptic Systems, Göttingen, Germany) overnight at 4 °C or stained with 0.02% methylene blue (MB) in PBS for 3 h and washed with ribonuclease-free water for 1 h as a reference for normalization. After incubation with HRP-conjugated anti-rabbit IgG secondary antibody (Invitrogen), the staining was visualized using a DAB Peroxidase Substrate Kit (Yeason, China).

### Lentivirus production and gene transduction

Lentiviruses were produced by cotransfecting individual shRNAs to suppress FTO expression using shRNA lentiviral particles (OBIO Technology, Shanghai, China), while noneffective scramble shRNA sequences (negative control, shNC) were used as the control group, with packing vectors (pLV-U6-[shRNA/shNC] CMV-EGFP-T2A-puromycin). To overexpress FTO in cells, lentiviruses were produced with the vectors (pLV-CMV-[FTO] PGK-EGFP-T2A-puromycin). Cells were infected following the instructions of the manufacturer and then incubated with 2 μg/mL puromycin for 72 h to select stable transfectants. The FOS siRNA and noneffective scramble siRNA sequences (negative control, siNC) were purchased from GenePharma Co., Ltd. (Shanghai, China) and transfected into cells with Lipofectamine 3000 (Invitrogen) according to the manufacturer’s instructions. The efficiency of downregulation of FTO or FOS in the cells was confirmed by RT-PCR and western blotting.

### Immunofluorescence staining and microscopy

The cells were cultured on 13-mm round glass coverslips (NEST, China). After the desired treatment, the cells were washed with cold PBS, fixed for 20 min in 4% paraformaldehyde, permeabilized, and blocked for an additional 30 min with Immunol Staining Blocking Buffer (Beyotime, China). Cells were subsequently incubated overnight at 4 °C with anti-FTO antibody (1:200; Abcam, Cambridge, USA), anti-c-FOS antibody (1:200; Affinity Biosciences, China), or anti-γHA. X antibody (1:200; Thermo Fisher Scientific) and incubated for 1 h with goat anti-rabbit IgG H&L (Abcam, Cambridge, USA) or goat anti-mouse IgG H&L (Abcam, Cambridge, USA). After washing five times in PBS and staining with DAPI (Beyotime, China) for 5 min, the coverslips were analyzed using an LSM 510 Zeiss laser confocal scanning microscope (Carl Zeiss, Oberkochen, Germany).

### β- galactosidase staining and microscopy

After washing and fixation, the cells were stained with a β-galactosidase Staining Kit (Beyotime, China; code No. c0602) according to the instructions. An anti-fluorescence quenching agent (Beyotime, China; code No. p0126) was used for sealing, and an LSM 510 Zeiss laser confocal scanning microscope (Carl Zeiss, Oberkochen, Germany) was used to take photos.

### RNA stability assay for mRNA lifetime

RNA stability assays were conducted as previously described [28] with the following modifications. Cells with stably expressed shRNA against FTO or shNC were seeded into 60 mm dishes to obtain ~70% confluency and were treated with 5 μg/ml actinomycin D 24 h later to inhibit RNA synthesis. The cells were then collected at the indicated time points to extract the total RNA and analyzed by RT-PCR as described above. The half-life and turnover rate of FOS mRNA was estimated according to a previously published paper [59] and our preexperiment.

### Luciferase reporter assays and mutagenesis assays

Three putative m6A recognition sites of the 3′UTR of FOS were identified in the 3′UTR by SRAMP [29]. Mutagenesis from A to C was generated by a QuikChange II Site-Directed Mutagenesis Kit (200, 523, Agilent, USA) according to the manufacturer’s instructions. The 3′UTR of FOS was amplified by PCR, which was cloned into the MCS restriction sites of pmiGLO (E133A, Promega, USA) to generate WT or MUT synthetic plasmids, and then the plasmids were transfected into the cells using Lipofectamine 3000 (Invitrogen). After 48 h, luciferase activity was measured by a Dual-Luciferase Reporter Gene Assay Kit (Yeason, China) with a multifunctional microplate reader. The Renilla luciferase activity values were normalized against the firefly luciferase activity values that reflect expression efficiency.

### Statistical analysis

#### For RNA sequence and MeRIP-seq

Briefly, paired-end reads were harvested from an Illumina HiSeq 4000 sequencer and quality controlled by Q30. After 3′ adapter trimming and low-quality read removal, cutadapt software (v1.9.3) was used. First, clean reads of all libraries were aligned to the reference genome (UCSC HG19) by Hisat2 software (v2.0.4). For the RNA m6A sequence, methylated sites on the RNAs (peaks) were identified by MACS software. Differentially methylated sites were identified by diffReps. These peaks identified by both software packages that overlapping with exons of the mRNA were determined and chosen by homemade scripts. GO and pathway enrichment analyses were performed for the differentially expressed and differentially methylated protein-coding genes.

#### Other analyses

All statistical analyses in this study were performed with GraphPad Prism 7.04, and image quantitative analyses were performed by using ImageJ 1.52a. Two-tailed Student’s *t*-tests were used to compare the significant differences between the indicated groups, while Pearson correlation analysis was used to analyze correlations. A *p* value <0.05 was considered significant.

## Supplementary information

Supplemental material
