# Correction: The m6A mRNA demethylase FTO in granulosa cells retards FOS-dependent ovarian aging

**DOI:** 10.1038/s41419-021-04194-6

**Published:** 2021-11-28

**Authors:** Zhong-xin Jiang, Yi-ning Wang, Zi-yuan Li, Zhi-hui Dai, Yi He, Kun Chu, Jia-yi Gu, Yi-Xuan Ji, Ning-xia Sun, Fu Yang, Wen Li

**Affiliations:** 1 Center for Reproductive Medicine & Fertility Preservation program, International Peace maternity and Child health Hospital, School of Medicine, Shanghai Jiao Tong University, Shanghai, 200030 China; 2 The Center of Reproductive Medicine, Shanghai Changzheng Hospital, Naval Medical University, Shanghai, 200003 China; 3 Obstetrics and Gynecology Department, The 1st Naval hospital of Southern Theater Command, Guangdong, 524005 China; 4 The Department of Medical Genetics, Naval Medical University, Shanghai, 200433 China

**Keywords:** RNA modification, Infertility

Correction to: *Cell Death and Disease* 10.1038/s41419-021-04016-9, published online 27 July 2021

The original version of this article unfortunately contained a mistake. Due to a typesetting error the affiliations were incorrect. We apologize for the mistake. The correct affiliations are:

Zhong-xin Jiang1, 2, 3, †, Yi-ning Wang2, †, Zi-yuan Li2, †, Zhi-hui Dai4, †, Yi He2, Kun Chu2, Jia-yi Gu2, Yi-Xuan Ji2, Ning-xia Sun2,*, Fu Yang4,*, Wen Li1, 2,*

Affiliations:

1 Center for Reproductive Medicine and Fertility Preservation Program, International Peace Maternity and Child Health Hospital, School of Medicine, Shanghai Jiao Tong University, 200030 Shanghai, China

2 The Center of Reproductive Medicine, Shanghai Changzheng Hospital, Naval Medical University, 200003 Shanghai, China

3 Obstetrics and Gynecology Department, The 1st Naval hospital of Southern Theater Command, 4 The Department of Medical Genetics, Naval Medical University, 200433 Shanghai, China524005 Guangdong, China

^†^These authors contributed equally.

* Corresponding author.

In addition, there was a misspelling in the footnote of Figure 1C: form should be changed to from. The original article has been corrected.

