# Supplementary material for: The m6A mRNA demethylase FTO in granulosa cells retards FOS-dependent ovarian aging
Source: Cell Death Dis. 2021 Jul 27;12(8):744. doi: 10.1038/s41419-021-04016-9 (PMC8316443; doi:10.1038/s41419-021-04016-9)
Supplement: Supplementary file 1 — Supplemental material [file 41419_2021_4016_MOESM1_ESM.docx]

Supplementary Materials for **The m6A mRNA demethylase FTO in granulosa cells retards FOS-dependent ovarian aging**

**This file includes:**

Supplementary Figure 1. **Results of Western Blots of the constructed cell lines**

Supplementary Figure 2. **Dot blot analysis of m6A modification in FTO knockdown GCs.**

Supplementary Figure 3. **Downregulating FOS was found in the two cell lines that overexpressed FTO, verified by RT-PCR.**

Supplementary Figure 4. **RIP-qPCR was performed with an FTO antibody.**

Supplementary Figure 5. **FOS siRNA was transferred into cells, verified by RT-PCR.**

Supplementary Figure 6. **Dot blot analysis of m6A modification in cells after incubation with hydrogen peroxide.**

Supplementary Table 1 **RNA adenosine methylation site prediction by SRAMP in FOS-mRNA**

Supplementary Table 2 **Eligibility and Exclusion Criteria**

Supplementary Table 3 **Clinical and biochemical profiles of patients with NOR and OA in this study**

Supplementary Table 4 **The STR sites of COV434 and KGN**

Supplementary Table 5 **Primers sequences used in RT-PCR**

Supplementary Table 6 **Antibodies used in this study**


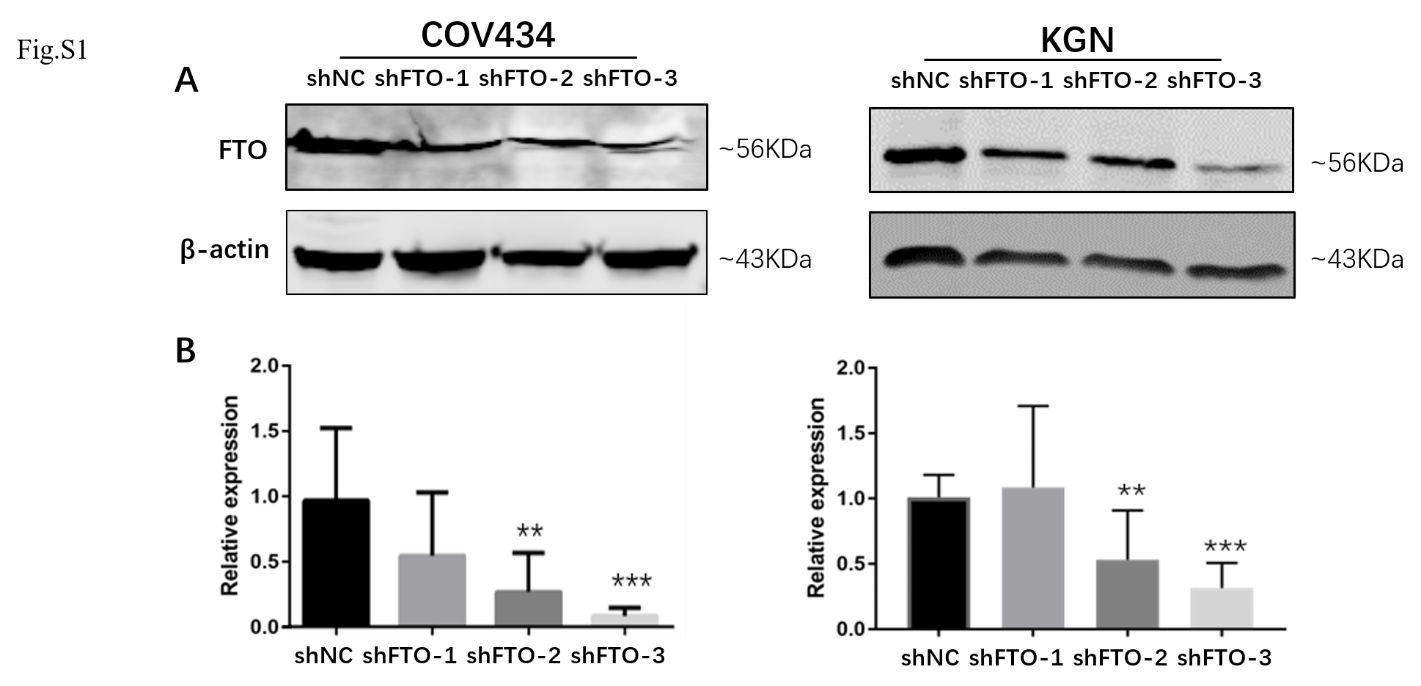


Fig. S1.

COV434 and KGN cell lines: COV434-shFTO, KGN-shFTO and their negative controls COV434-shNC and KGN-shNC were constructed by 3 kind of lentivirus, which were verified by western blot (A) and RT-PCR (B).


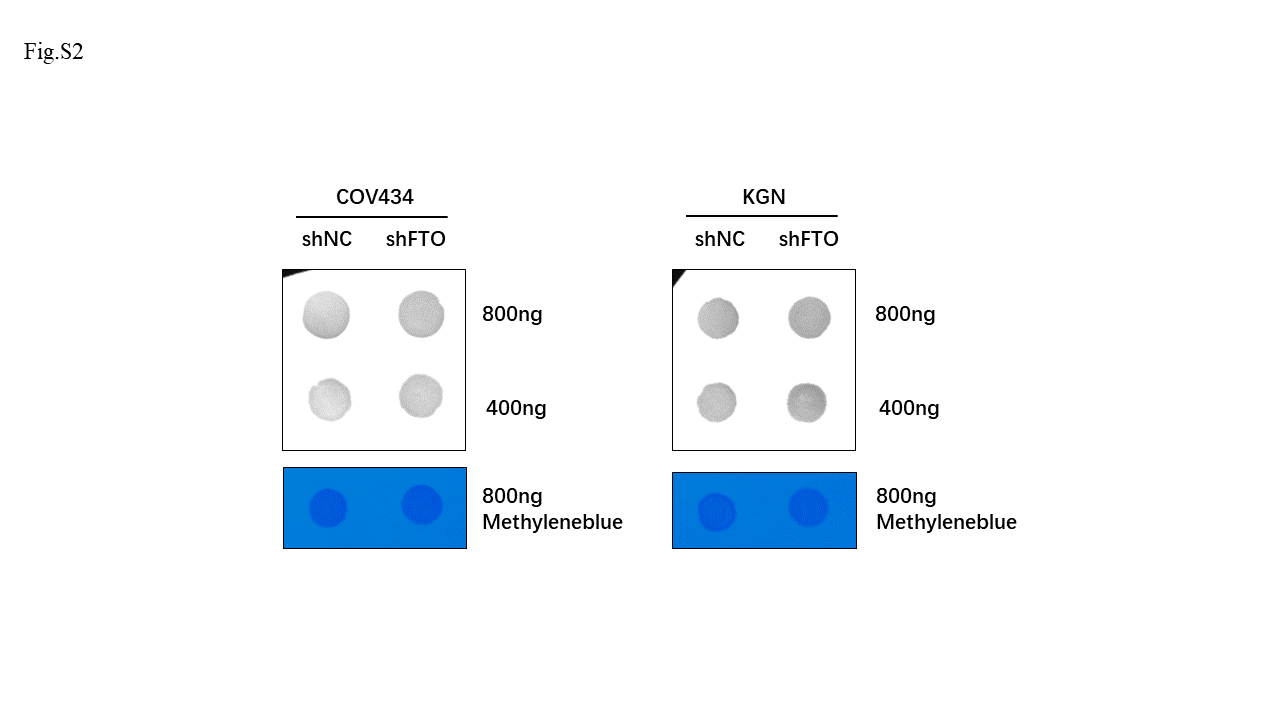


Fig. S2.

Dot blot analysis showed increased m6A modification in FTO knockdown GCs.


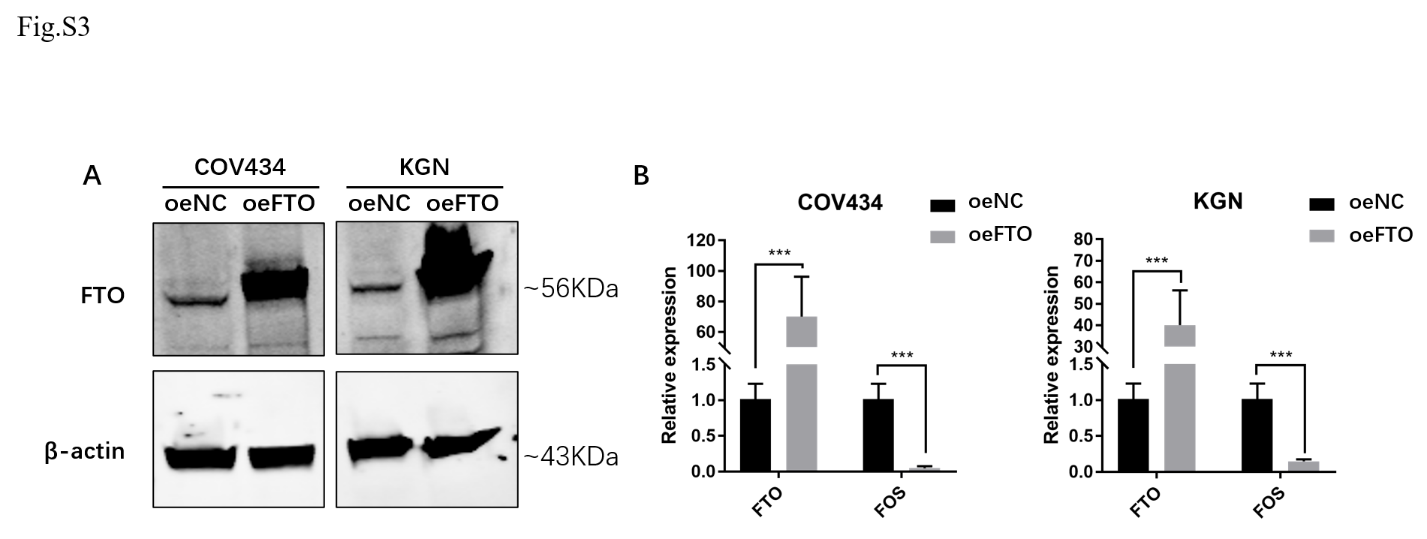


Fig. S3.

FOS was found to be significantly downregulated in the two cell lines that overexpressed FTO, which were verified by western blot (A) and RT-PCR (B).


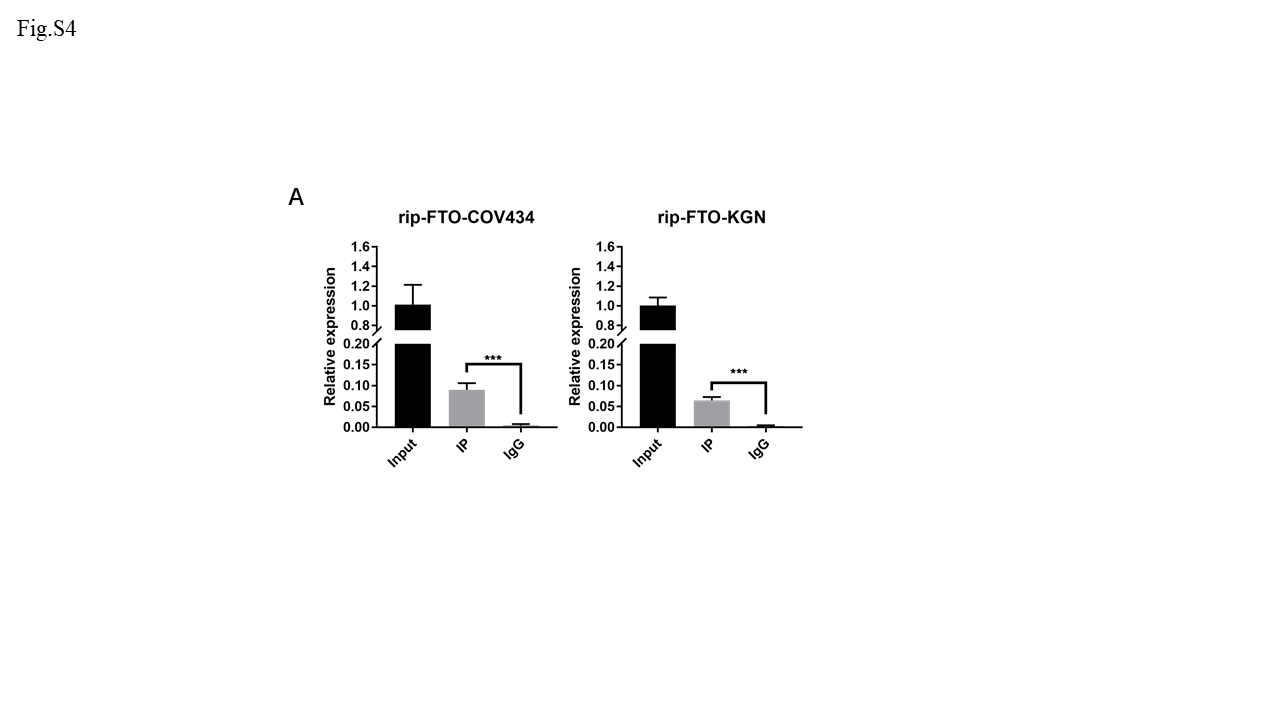


Fig. S4.

RIP-qPCR was performed with an FTO antibody and we found that the FTO protein could bind to FOS mRNA.


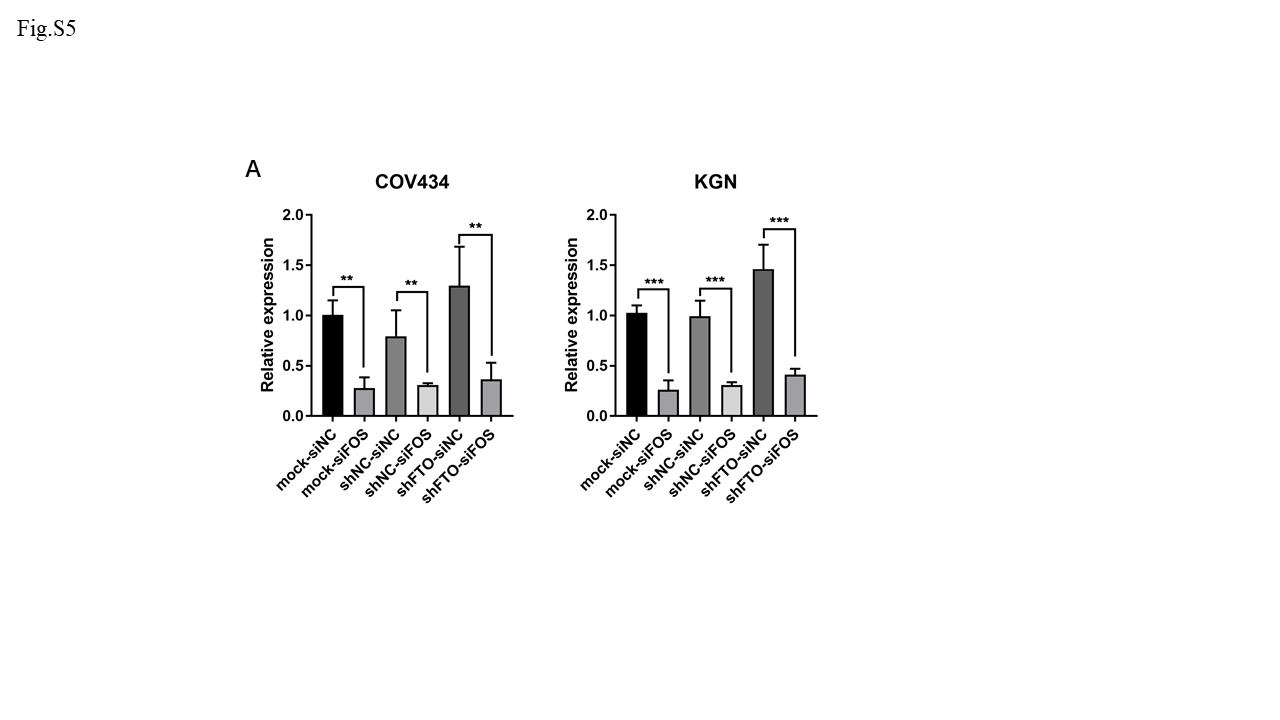


Fig. S5.

FOS siRNA was transferred into COV434 and KGN cells before and after FTO knockdown, verified by RT-PCR.


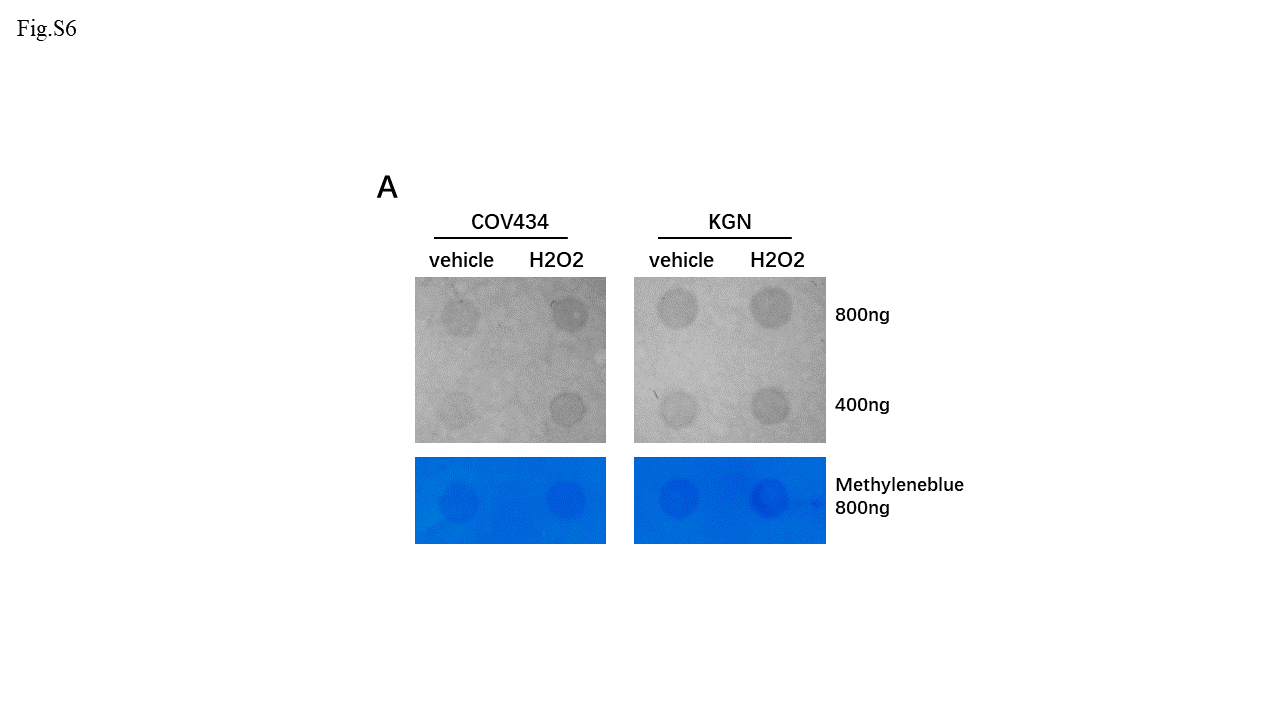


Fig. S6.

The m6A modification of total RNA significantly increased after incubation with hydrogen peroxide.

Table S1.

RNA adenosine methylation site prediction by SRAMP in FOS-mRNA.

**Table S1. RNA adenosine methylation site prediction by SRAMP in FOS-mRNA**

| Position | Sequence context | Structural context | Score(binary) | Score(knn) | Score(spectrum) | Score(combined) | Decision | # |
| --- | --- | --- | --- | --- | --- | --- | --- | --- |
| 35 | AGCGAGCAUCUGAGAAGCCAAGACUGAGCCGGCGGCCGCGGCGCA | PIIPPPPMMMMMMPPPPPPPPPPPMMMPPIPPPPPMMPBPPPPPI | 0.675 | 0.742 | 0.586 | 0.643 | m6A site (High confidence) | 1 |
| 181 | AUGUUCUCGGGCUUCAACGCAGACUACGAGGCGUCAUCCUCCCGC | PPPPMPPPPIPPHHHHHHPPIIIIIIPPPPPPPPPPPPMMMMMPP | 0.569 | 0.711 | 0.593 | 0.586 | m6A site (Moderate confidence) | 2 |
| 259 | UCUUACUACCACUCACCCGCAGACUCCUUCUCCAGCAUGGGCUCG | MMMMMMMMMMMMMMMMMMPPPPMMMMMMMMMMMMPPPPPPPMMMM | 0.463 | 0.673 | 0.644 | 0.546 | m6A site (Low confidence) | 3 |
| 298 | GGCUCGCCUGUCAACGCGCAGGACUUCUGCACGGACCUGGCCGUC | PPPPPPPPIPPPPBPIIPPPPPMPHHHHPMMPPPPPIPPPIPPII | 0.739 | 0.686 | 0.605 | 0.683 | m6A site (Very high confidence) | 4 |
| 310 | AACGCGCAGGACUUCUGCACGGACCUGGCCGUCUCCAGUGCCAAC | PBPIIPPPPPMPHHHHPMMPPPPPIPPPIPPIIIIPPPPPPPHHH | 0.601 | 0.585 | 0.603 | 0.601 | m6A site (High confidence) | 5 |
| 370 | ACUGCCAUCUCGACCAGUCCGGACCUGCAGUGGCUGGUGCAGCCC | PPPPIIIIIIPPMPPPPPPPPMMPPPPPIPPPPPIPPPPBPPPPP | 0.523 | 0.545 | 0.575 | 0.545 | m6A site (Low confidence) | 6 |
| 495 | CUCCAGGGCUGGCGUUGUGAAGACCAUGACAGGAGGCCGAGCGCA | PPPPPPPPPPPPIIPPPPPPIPIPPPPPIIPPPPPPBPPPPIPPM | 0.386 | 0.586 | 0.763 | 0.547 | m6A site (Low confidence) | 7 |
| 501 | GGCUGGCGUUGUGAAGACCAUGACAGGAGGCCGAGCGCAGAGCAU | PPPPPPIIPPPPPPIPIPPPPPIIPPPPPPBPPPPIPPMMMPPBB | 0.422 | 0.528 | 0.765 | 0.564 | m6A site (Moderate confidence) | 8 |
| 545 | UUGGCAGGAGGGGCAAGGUGGAACAGUUAUCUCCAGAAGAAGAAG | BPPPBPPPPPPPPIIIIPPPPMMMMMMMMPPPPHHHHHHHHHHHP | 0.511 | 0.486 | 0.78 | 0.617 | m6A site (High confidence) | 9 |
| 639 | CCGCAACCGGAGGAGGGAGCUGACUGAUACACUCCAAGCGGAGAC | MMPPPPPPPPIIPPPPPBPPPPPMPPHHHHHHHHPPMPPPPPPPP | 0.593 | 0.642 | 0.648 | 0.617 | m6A site (High confidence) | 10 |
| 731 | ACCUGCUGAAGGAGAAGGAAAAACUAGAGUUCAUCCUGGCAGCUC | MPPPHHHHHPPPMMMPPPPIIPPPHHHHPPPIIPPPPPPPPPPPP | 0.496 | 0.33 | 0.7 | 0.569 | m6A site (Moderate confidence) | 11 |
| 825 | GUCUGUGGCUUCCCUUGAUCUGACUGGGGGCCUGCCAGAGGUUGC | PPMMMMPPPPPPPMMMPPPPPPIIIPPPPPPIIPPPHHHPPPIPP | 0.528 | 0.638 | 0.646 | 0.581 | m6A site (Moderate confidence) | 12 |
| 1033 | GCCCGCUCCGUGCCAGACAUGGACCUAUCUGGGUCCUUCUAUGCA | PPPMPPPPPPPPPMPPPPMMPPPPPPHHHHPPPPPPMMMMMMPPP | 0.868 | 0.846 | 0.858 | 0.863 | m6A site (Very high confidence) | 13 |
| 1060 | UCUGGGUCCUUCUAUGCAGCAGACUGGGAGCCUCUGCACAGUGGC | HHHPPPPPPMMMMMMPPPPPPPBPPPPPPPMMMMPPPPPPPIPPP | 0.544 | 0.774 | 0.598 | 0.577 | m6A site (Moderate confidence) | 14 |
| 1411 | GGUUCCUGUAGACCUAGGGAGGACCUUAUCUGUGCGUGAAACACA | PPPPHHHHHHPPPPPPPPPPPPPPPPPIPPPPPPIPPPBBBBBPP | 0.524 | 0.549 | 0.553 | 0.537 | m6A site (Low confidence) | 15 |
| 1454 | CACCAGGCUGUGGGCCUCAAGGACUUGAAAGCAUCCAUGUGUGGA | PPPPPMPPPMMMPPPPPPMPPPPPPPPPIIIIIPPPPHHHHPPPP | 0.898 | 0.734 | 0.822 | 0.859 | m6A site (Very high confidence) | 16 |
| 1476 | ACUUGAAAGCAUCCAUGUGUGGACUCAAGUCCUUACCUCUUCCGG | PPPPPPIIIIIPPPPHHHHPPPPMPPPPPPPPPMMMMMMMMMMPP | 0.64 | 0.67 | 0.392 | 0.542 | m6A site (Low confidence) | 17 |

Table S2.

Eligibility and Exclusion Criteria.

| **Table 2.** **Eligibility and Exclusion Criteria.** |
| --- |
| **Basic inclusion criteria** |
| 1. Over 18 years old and under 50 years old |
| 2. Clear consciousness, able to provide accurate information |
| 3. Informed consent signed  4. More than two oocyte-granulosa cell complexes can be obtained by superovulation  5. No ovarian operation, radiotherapy or chemotherapy has been carried out that affect the ovarian function, and no chromosome abnormality was found |
| **Normal ovarian reserve (NOR group)** |
| 1. Infertility caused by male factor or famale fallopian tube factor |
| 2. Age < 37  3. AMH ≥ 1.1ng/dl  4. AFC ≥ 5 |
| **Ovarian aging (OA group)** **^†^** |
| 1. Infertility caused by female ovarian dysfunction  2. Age ≥ 37  3. AMH < 1.1ng/dl  4. AFC: 2~4 |

^†^ Reference to the Bologna criteria for poor ovarian response (Bologna criteria) published by the European Society of Human Reproduction and Embryology (ESHRE) in 2011 and clinical guidelines for Premature ovarian insufficiency published in 2016.

Table S3.

Clinical and biochemical profiles of patients with NOR and OA in this study.

**Table 3.** **Clinical and biochemical profiles of patients with OA and NOR in this study**

| **Variable** | **NOR** | **OA** |
| --- | --- | --- |
| Age (years) | 28±1.90 | 42.12±3.80 |
| BMI (Kg/m2) | 20.26±0.63 | 23.00±1.86 |
| Basal LH(IU/L) | 2.64±0.86 | 3.79±1.46 |
| Basal FSH(IU/L) | 3.65±1.52 | 10.36±3.22 |
| AMH (ng/mL) | 3.80±1.01 | 0.66±0.36 |
| Hormones on hCG day E_2_(pg/mL) | 3342±682.24 | 596.80±296.25 |

Table S4.

**The STR sites of COV434 and KGN**.

COV434 KGN


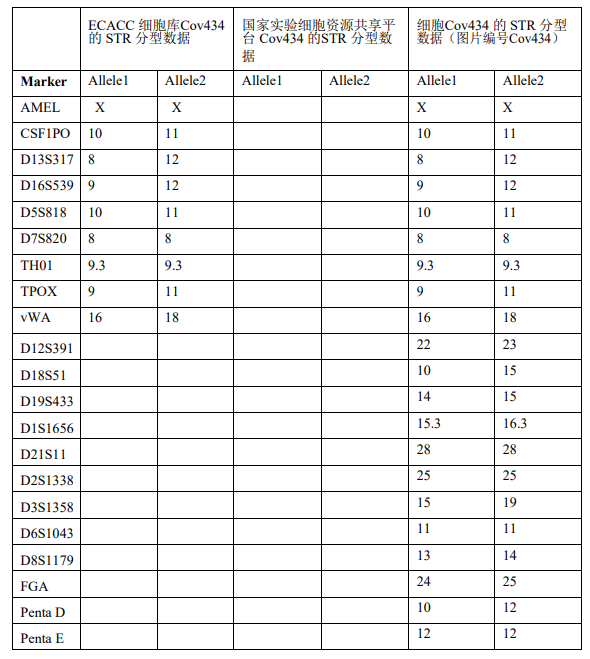

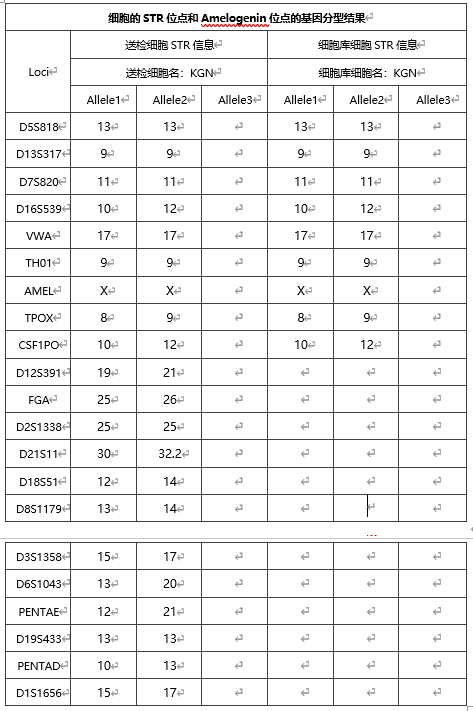


Table S5.

Primers sequences used in RT-PCR.

**Table 3.** **Primers sequences in RT-PCR**

| Primer Name | Sequence （5'-3'） |
| --- | --- |
| GAPDH-F | CGGATTTGGTCGTATTGGG |
| GAPDH-R | CTGGAAGATGGTGATGGGATT |
| FTO-F | TGGGTTCATCCTACAACGG |
| FTO-R | CCTCTTCAGGGCCTTCAC |
| ALKBH5-F | CCCGAGGGCTTCGTCAACA |
| ALKBH5-R | CGACACCCGAATAGGCTTGA |
| METTL3-F | CTGCTGATTTCTCAACCT |
| METTL3-R | CTGGCTATTTACTTGATTCATT |
| METTL4-F | TAAAGACGGCAAGACCAT |
| METTL4-R | TGATGCCAGGAAAGAACA |
| METTL14-F | AGAAACTTGCAGGGCTTCCT |
| METTL14-R | TCTTCTTCATATGGCAAATTTTCTT |
| WTAP-F | GGCGAAGTGTCGAATGCT |
| WTAP-R | CCAACTGCTGGCGTGTCT |
| KIAA1429-F | GAATACTTGATGGTCTGGTGCTA |
| KIAA1429-R | CTTGGCTGTGGTCTTGGA |
| FOS -F | GGGGCAAGGTGGAACAGTTAT |
| FOS-R | CCGCTTGGAGTGTATCAGTCA |
| NEDD9-F | CTACAGGGTAAGGAGGAGTTT |
| NEDD9-R | TGAAGAGTGCGTCAATGG |
| CCDC80-F | GACCCCGTTTCACTATGCTGT |
| CCDC80-R | GGCGAGCTAGTCTCAACACG |
| LIN52-F | CTAGTTCTCCACCCAAATGGATG |
| LIN52-R | GCTGATAGGCTAGGTTCTGTAGG |
| COL6A2-F | CCGGGGATAGCCTCTCTTACT |
| COL6A2-R | CTTGTAGCACTCTCCGTAGGC |
| PLCG2-F | TCCACCACGGTCAATGTAGAT |
| PLCG2-R | CCCTGGGCGGATTTCTTTTAT |
| ZBTB3-F | TCCTTCGTGGGGTACTATGGA |
| ZBTB3-R | CCAATTCCCGCTCCTTGTAGAA |
| CDK6-F | CCAGATGGCTCTAACCTCAGT |
| CDK6-R | AACTTCCACGAAAAAGAGGCTT |
| LRRC26-F | GCCGCCTTTAGCCATTG |
| LRRC26-R | TCGGGGTTCGGGTCTG |
| RBM47-F | GCCGCCCAATAACTCCAGTAT |
| RBM47-R | CGTTCTTCTGTAGTGTGGCGA |
| HES7-F | CGGGATCGAGCTGAGAATAGG |
| HES7-R | GCGAACTCCAATATCTCCGCTT |

Table S6.

Antibodies used in this study.

**Table 5.** **Antibodies used in this study**

| Antibody | Supplier | Catalogue number | Primary/  secondary antibody | Host | Mono-/polyclonal |
| --- | --- | --- | --- | --- | --- |
| Anti-m6A | SySy | 202011 | P | rabbit | p |
| FTO | Abcam | Ab126605 | P | rabbit | m |
| γH2A.X | ThermoFisher | 14-9865-82 | P | mouse | p |
| FOS | Abcam | Ab208942 | P | mouse | m |
| β-actin | Proteintech | 66009-I-1g | P | mouse | m |
| Anti-Rabbit IgG (H+L) Secondary Antibody, HRP | Invitrogen | 65-6120 | S | goat | p |
| IRDye Goat anti-Mouse 680RD | LI-COR | 926-68070 | S | goat | p |
| IRDye Goat anti-Rabbit 800CW | LI-COR | 926-32211 | S | goat | p |
